# Closing the Gender Gap: The Case for Gender-Specific Alcoholism Research

**DOI:** 10.4172/2329-6488.1000e106

**Published:** 2013-08-27

**Authors:** Susan Mosher Ruiz, Marlene Oscar-Berman

**Affiliations:** 1 Department of Anatomy & Neurobiology, Boston University School of Medicine, USA; 2 Psychology Research Service, VA Boston Healthcare System, USA; 3 Behavioral Neuroscience Program, Boston University School of Medicine, USA

**Keywords:** Alcoholism, Gender, Alcohol, Brain, Women, Neuroimaging

## Abstract

As the number of women who use alcohol increases, so does the number of women who engage in alcohol abuse and develop alcohol dependence. The recent increased focus on women and gender differences in alcoholism research has largely come about following recognition that the face of alcoholism is changing, with alcoholism rates among men remaining stable and rising among women, particularly in younger women. As such, the need to understand gender differences in both acute and long-term effects of alcohol abuse has never been more critical.

Gender differences in the long-term effects of chronic alcoholism on the brain and other systems are currently under debate, often with a focus on proclaiming whether men or women suffer the most impact. However, the story appears to be more complex than that. The issue of how alcoholism interacts with gender is complicated, as gender differences in many factors including alcohol metabolism, alcoholism progression, problematic drinking patterns, neurobiology, hormones, and psychiatric comorbidities will contribute to the differences in structural and functional outcomes observed experimentally across domains of inquiry. While women are now much more commonly included in studies of alcohol’s effects on the brain, there remains a need for more explicit examinations of gender effects.

## Editorial

Historically, across many cultures, drug and alcohol addiction were considered to be diseases that affected men only. Currently in the United States, it is estimated that alcoholism rates among men are more than twice as high as they are in women (5.5% in men versus 1.9% in women) [1]. Additionally, current alcohol use rates in the US are considerably lower among women than among men, with approximately 43% of women and 61% of men reporting current alcohol use [2]. In part, this demonstrates why so much of the body of research on alcoholism has focused on men. However, the traditional gender gap in alcohol use and abuse rates is closing. Among younger adults, current alcohol and other drug abuse rates are reported to be equivalent between men and women [3,4], with some reports even showing higher rates of alcohol use among girls in the youngest cohorts [5]. While the number of women who fit research criteria for alcohol abuse is increasing, research focusing on long-term and acute consequences of alcohol consumption specifically in women remains relatively rare. In part due to NIH mandates, exclusion of women as research participants is less commonplace [6]. However, mixed-gender examinations of alcohol’s effects on brain structure and function often fail to address gender effects explicitly. Neglecting consideration of gender as a factor in alcoholism studies is unfortunate, as there are well-demonstrated gender differences in alcohol metabolism, progression toward alcoholism, drinking patterns, neurobiology, and comorbid mood disorders. Given compelling evidence for the many factors that could potentially contribute to observable differences in alcohol’s effects on the brain between men and women, the need to address the question of gender in alcoholism outcomes is crucial.

Physiologically, gender differences exist in how alcohol is processed in the body, including the central nervous system [7,8]. Women have lower activity of alcohol dehydrogenase in their stomachs, which leads to higher peak blood alcohol concentrations (BAC) in women than in men with the same amount of alcohol consumed. Women also tend to be smaller and to have lower volumes of water in their bodies than men, further contributing to higher peak BACs in women [8]. However, it has been suggested that ‘alcohol disappearance’ rates (*i.e.*, rates of decrease from peak BAC) are slower among men [9]. Because alcohol is miscible with water, it easily crosses the blood-brain barrier. Since the brain is well perfused relative to other structures in the body, alcohol concentrations there will be higher than in venous blood taken from the muscles [10]. As such, differences in BACs achieved across genders will be pronounced in the brain. These gender-based differences in alcohol metabolism render disparities in measures of daily alcohol consumption difficult to interpret, and suggest that examinations of consumption variables might be better understood by examining men and women separately.

One of the earliest reports of gender differences among alcoholics was the description of the telescoping effect [11], wherein alcoholic women were found to initiate alcohol use at a later age, progress through the stages of alcoholism more quickly, and display physical symptoms of alcoholism with shorter durations of alcohol abuse than men [12,13]. The phenomenon that women more rapidly escalate substance use also has been reported for a number of other drugs, including opiates, marijuana, and cocaine [14]. Recent studies examining the telescoping effect in alcoholism have confirmed a more rapid progression of alcoholism among women [15,16]. However, one study that failed to find accelerated progression of alcoholism among women also reported that women in younger cohorts were initiating drinking and becoming dependent at ages closer to those observed in men [17]. Also in accordance with diminishing patterns of gender differences in alcohol abuse rates, a study that confirmed a generalized telescoping effect examined age effects of telescoping and found gender differences in alcoholism progression to be attenuated among younger adults relative to older adults [13]. Taken together, these findings suggest that gender differences in the development of alcohol use disorders were historically more likely to involve faster progression among women, but that among younger adults, this difference is less common. This highlights the need for addressing cohort effects when examining gender differences in alcoholism [18].

Gender differences in drinking behaviors (*e.g.*, pace of drinking, drinks per session, types of drinks consumed) are numerous and are likely to contribute to differential outcomes between genders following years of alcohol abuse [19,20]. The rapid progression of alcoholism that has been commonly observed in women in older cohorts may explain the typically shorter durations of problem drinking that have been observed in women relative to men. In terms of consumption, in nonhuman animal models, females have been found to consume higher levels of alcohol than males [21], whereas in humans, the opposite pattern typically is observed [19]. However, among adolescent drinkers, gender differences in quantities of alcohol consumed are becoming small [22]. As there exists a wealth of evidence for recovery of brain tissue and functioning, gender-based differences in abstinence and treatment patterns [23] further contribute to the need for examining alcoholic men and women independently.

In addition to complex alcohol metabolism considerations and dimorphic drinking patterns, gender differences in neurobiology also will influence the effects of acute and chronic alcohol administration [24]. During typical alcohol administration, release of serotonin, dopamine, and GABA are stimulated, and glutamatergic neurotransmission is blocked [25]. Gender differences have been reported in striatal dopaminergic function [26,27], GABA [28] and N-acetylaspartate [29] concentrations, as well as NMDA receptor expression [30], all of which are likely to influence differential patterns of vulnerability to alcohol dependence and reward system functioning.

The influence of gonadal and stress hormones also contributes to the differences observed in the brains of alcoholic men and women, particularly in terms of the central nervous system adapting to chronic alcohol administration and withdrawal [31–33]. These hormones have been shown to modulate the action of reward circuitry in particular [34]. Besides differences between the sexes, within the population of just alcoholic women, differences in menstrual cycles and the use of hormonal contraceptives and hormone replacement therapy can influence alcohol metabolism and neurobiology [35], and assessment of these influences are scarce.

Further, gender differences in rates of mental disorders are consistently reported in epidemiological studies, with mood disorders (*e.g.*, anxiety, depression) more common among women, and externalizing disorders (*e.g.*, ASPD, conduct disorder, ADHD) more common among men [4,36–38]. These disorders have high comorbidity with alcoholism, and their prevalence across genders in alcoholism exhibit complex interactions with behavioral factors such as treatment and dependence severity [39]. These comorbid mood disorders have been shown to interact with alcoholism to affect neurocognitive functioning [40].

All of these factors will contribute to differences between genders in the patterns of cognitive and emotional dysfunction and neural tissue damage that have been observed in alcoholism. Evidence from nonhuman animal research has suggested that females may be more susceptible to alcohol-induced brain damage [41]; however, research describing gender differences among human alcoholics in brain structure and function has been equivocal [42–44]. Direct comparisons of alcoholic men and women in functional imaging are relatively rare, and brain structural analyses have yielded conflicting results across studies. Disparate findings are further complicated by the fact that many of the factors described above that can influence both structural and functional outcomes in alcoholism have gender-based differences that have not been taken into consideration [42]. Changes in brain tissue in long-term chronic alcoholism can vary not only with gender, but also with age, quantity of alcohol consumed, and duration of alcoholism period. Across studies, different subsets of these confounding variables are controlled for [4,45], and interactions of each of these factors with gender remain to be well studied [46].

In sum, it is clear that from the age at their first drink, through evolution to alcohol dependence, and on to treatment and recovery, alcoholic men and women tend to follow different paths. Many factors contribute to the observed differences in neural structure and function between alcoholic men and women. Biology plays a large role, with gender differences in alcohol metabolism, neurobiology, and hormones contributing to a complex web of interrelated physiological processes, all of which ultimately influence measurable functional and structural outcomes. Further, it is becoming better understood that gender effects can vary with many other factors, including age, race, diet, and type and quantity of alcohol consumed. Given all the factors that contribute to the differences that are observed empirically, gender effects may be masked or revealed depending on which factors are controlled for. Assessing these differences presents methodological challenges, and highlights the need for multimodal approaches to understanding gender-based differences using neuroimaging. Given the many factors that differ between alcoholic men and women, inclusion of gender-based analyses of neuroimaging data is imperative. Ultimately, an understanding of gender differences in behavioral patterns, neural structure and function, and physiology between alcoholic men and women will lead to the development of prevention, intervention, and treatment programs that are tailored to each gender’s unique needs.

## Key Points

Alcohol use, abuse, and dependence rates are rising among women.

There are many factors that differentiate men and women in terms of their profiles as alcoholics; these all contribute to observed gender differences.

More research is needed to determine how these factors contribute to gender differences (or lack thereof) in outcomes.

## Abbreviations

ADHD: Attention Deficit Hyperactivity Disorder
ASPD: Antisocial Personality Disorder
BAC: Blood Alcohol Concentration
GABA: *Gamma*-Aminobutyric acid
NIH: National Institutes of Health
NMDA: N-methyl-D-aspartate
